# The Dissemination of a Single *Staphylococcus* *aureus* Strain, *Spa*-t2873, as the Predominant Cause of Bovine Mastitis in Israeli Dairy Farms

**DOI:** 10.3390/vetsci9070371

**Published:** 2022-07-19

**Authors:** Adin Shwimmer, Rama Falk, Tamar K. De-Levie, Michal Lasnoy, Katia Levytskyi, Amos Adler

**Affiliations:** 1 National Service for Udder Health and Milk Quality, Israeli Dairy Board, Caesarea 38900, Israel; adin@milk.org.il (A.S.); rama@milk.org.il (R.F.); 2 Sackler Faculty of Medicine, Tel Aviv University, Tel Aviv 69978, Israel; tamarkope@gmail.com; 3 Clinical Microbiology Laboratory, Tel Aviv Sourasky Medical Center, Tel Aviv 64239, Israel; michallas@tlvmc.gov.il (M.L.); katiaza@tlvmc.gov.il (K.L.)

**Keywords:** *Staphylococcus aureus*, bovine mastitis, transmission, whole-genome sequencing

## Abstract

**Simple Summary:**

*Staphylococcus aureus* is one of the most important pathogens causing intramammary infection (IMI) in cattle. We aimed to characterize which are the specific strains that are responsible for the majority of these cases. During the one-year survey, *S. aureus* was identified in 152 dairy farms, with a total of 440 positive samples. One specific type (designated-*spa* t2873) was found in 284 isolates (64.5%). Notably, 25 cases were detected in one specific farm, all of which were found to be dominant *spa* t2873 type. By using genomic analysis, we were able to ascertain that most transmission events occurred within the same milking group. Our study revealed the dissemination of a single strain to the majority of Israeli dairy farms. The possibility of inter-farm transmission should be monitored and prevented.

**Abstract:**

*Staphylococcus aureus* is one of the most important pathogens causing intramammary infection (IMI) in dairy herds. The goals of this study were (1) to describe the prevalence of *S. aureus* in Israeli dairy farms; (2) to characterize the *spa*-based clonal structure of mastitis-related *S. aureus* isolates; (3) to analyze the transmission network of a large outbreak within a single farm and (4) to characterize the virulence factors of the outbreak strain. The prevalence and the molecular survey were performed on all Israeli IMI-related isolates, 9.2019–8.2020. Molecular methods included *spa*-typing for the survey and whole-genome sequencing (WGS) for the investigation of the farm ‘A’ outbreak. During the one-year survey, *S. aureus* was identified in 152 dairy farms, with a total of 440 positive samples. The *spa* t2873 was found in 284 isolates (64.5%) across 112 farms (73.6%). Other common types included t529 (n = 46), t9303 (n = 34) and the methicillin-resistant *S. aureus* t011 (n = 11). The highest number of cases (n = 25) was detected in Farm ‘A’, all of which were found to be *spa* t2873. Phylogenetic analysis confirmed that most transmission events occurred within the same milking group, and inter-group transmission was due to the transfer of cows between groups or due to consecutive milking order. The *spa* t2873 strain contained putative virulence genes, including various intracellular and collagen adhesion proteins. Our study revealed the dissemination of the t2873 strain to the majority of Israeli dairy farms. The possibility of inter-farm transmission should be monitored and prevented.

## 1. Introduction

*Staphylococcus aureus* is globally one of the most important pathogens causing clinical and sub-clinical mastitis in dairy herds. *S. aureus* often spreads during the milking process, causing alveolar tissue damage and reduced milk quality and production, leading to substantial economic losses in the dairy industry [1].

In the recent decade, substantial interest was spent on one strain, the methicillin-resistant *S. aureus* (MRSA) clonal complex (CC) 398, that had spread globally in bovine and other livestock animals [2]. This strain was identified in Israel and caused an outbreak on one farm [3], but the overall rate of MRSA had remained low. Despite the low prevalence of MRSA, the burden of *S. aureus* mastitis has been increasing in recent years [3].

Although changes in the incidence of mastitis might be due to risk factors related to milking practices or prevention measures [1], one possible explanation might be the expansion of a transmissible strain. Such phenomenon is well known in both livestock and human *S. aureus* infections [4]. Thus, an important method to elucidate the possibility of an emerging *S. aureus* would be the conduction of a national survey of all *S. aureus* isolated from milk cultures. Of note, recent national surveys of *S. aureus* mastitis isolates that were conducted in other countries [5,6,7] showed diverse clonal structures with more than a single dominant clone.

The goals of this study were (1) to describe the prevalence of *S. aureus* in Israeli dairy farms from 1 September 2019 until 31 August 2020; (2) to characterize the *spa*-based clonal structure of mastitis-related *S. aureus* isolates in an annual survey in Israel; (3) to analyze the transmission network of a large outbreak within a single farm and (4) to characterize the virulence factors of the outbreak strain.

## 2. Methods

### 2.1. Study Design

The study included three parts: (1) a microbiological-molecular national survey of mastitis-related *S. aureus* isolates; (2) a molecular epidemiology study tracking the dissemination of the t2873 in farm ‘A’ (see below); (3) a genomic study of the molecular features of the t2873 strain (see below).

The national survey underwent from 1 September 2019 until 31 August 2020 and included all *S. aureus* isolates (first isolate per cow) that were cultured from milk samples that were collected as part of routine animal care. Milk samples were submitted to the Laboratory for Udder Health and Milk Quality (UHL), an ISO17025-certified laboratory that provides diagnostic services to all dairy farms in Israel. All samples were sent voluntarily. Isolates in the study originate from (1) cows exhibiting high-somatic cell counts (HSCC), as based on the monthly Dairy Herd Improvement (DHI) results (approximately 45% of the isolates); (2) cows exhibiting clinical manifestations (approximately 42% of the isolates); (3) cows before dry-off treatment and after-calving (approximately 13% of the isolates). As the study included microbiological analysis of isolates and did not involve any intervention in routine animal care, Ethical Committee approval was not required. 

### 2.2. Microbiological Methods and Spa Typing

Milk samples, both individual and bulk tank samples, were sent from participating farms to the UHL. A total of 10 µL of milk samples were streaked on 5% sheep blood Tryptic Soy Agar plates and incubated at 37 °C for 48 h in accordance with the National Mastitis Council [8]. *S. aureus* was identified by using typical morphology and biochemistry characteristics, i.e., positive catalase and coagulase tests (Coagulase plasma rabbit, BD). *S. aureus* isolates were sent to the Tel Aviv Sourasky Medical Center (TASMC) microbiology laboratory for further testing. Antimicrobial susceptibility testing was performed by the disk diffusion test (DDT) for the following antimicrobials: cefoxitin, doxycycline, ciprofloxacin, gentamicin, clindamycin, erythromycin, rifampin, trimethoprim-sulfamethoxazole, and linezolid. The *Spa* gene PCR and sequencing were used for species confirmation, and typing was performed and analyzed as previously performed [9].

### 2.3. Next-Generation Sequencing and Analysis

Whole-genome sequencing (WGS) was performed as previously described [10] using the Illumina MiSeq system. In brief, libraries were prepared using Nextera DNA Flex Library Prep Kit (Illumina, Inc., San Diego, CA, USA) according to the manufacturer’s instructions. After sequencing each library, FASTAQ files were imported into CLC Genomics Workbench v.12.0.3 (Qiagen, Denmark) included, and the reads were de novo assembled into contigs. Illumina reads were mapped to a reference genome (NC_007795-1) and annotated using the Microbial Genomic Module of the CLC Workbench software. Phylogenetic analysis was performed by SNP calling for spa t2873 isolates using the CLC Workbench software [11,12] of all shared SNPs with coverage of ≥10, for a total input of 105 SNPs. Phylogenetic tree was constructed using the Neighbor-Joining algorithm and included the metadata of isolation date and milking group. Detection and characterization of resistance and virulence genes were performed by the Find Resistance with Nucleotide DB tool of the CLC software.

### 2.4. Epidemiological Study of the t2873 Strain Transmission in Farm ‘A’

The aim of this part of the study was to elucidate the transmission of the t2873 strain inside a farm. Farm ‘A’ was chosen as this was the farm with the largest number of positive samples (n = 27) identified in the national survey. The epidemiological investigation was conducted through two site visits and collection of data regarding the basic structure and characteristics of the farm (including milking practices) and data regarding the whereabouts of each of the afflicted cows before and after the date of *S. aureus* isolation. The epidemiological data were aligned with the WGS data in order to draw a putative transmission network.

## 3. Results

### 3.1. S. aureus Prevalence in Israeli Dairy Farms

*S. aureus* was identified in 1.7% of all milk samples, whereas other Staphylococci species were identified in 22% of the samples. *S. aureus* was identified in 152 farms out of the 547 dairy farms (27.7%) that submitted milk samples to the UHL during the one year of the study (September 2019–August 2020). In the 152 *S. aureus* infected farms, there were a total of 440 positive samples, of which 394 were individual samples, and 46 were bulk tank samples. The two most common indications that were registered for the individual samples were clinical infection (n = 165), and high individual cow somatic cell count (ICSCC) measured on the monthly DHI (n = 180).

### 3.2. Molecular and Microbiological Features of S. aureus Isolates

The distribution of *S. aureus* isolates according to *spa* types is presented in Table 1. The MSSA *spa* t2873 was found in 284 isolates (64.5%) across 112 farms (73.6% of afflicted farms), in both individual and tank samples. As with all isolates, the common indications for individual samples were clinical infection (n = 126) and high somatic cell count (SCC) (n = 100). Other common types (n > 10) included the MSSA t529 (n = 46), t9303 (n = 34), t1250 (n = 13) and the MRSA t011 (n = 11), that was identified in three farms. The rate of MRSA was very low (13/440, 3%), with other MRSA types including t034 (n = 1, out of 8 MSSA isolates) and t2346 (n = 1). The overall susceptibility to antimicrobial was very high, with susceptibility rates to doxycycline, ciprofloxacin, gentamicin, clindamycin, erythromycin, rifampin, trimethoprim-sulfamethoxazole, and linezolid of 63.4%, 95.6%, 97.7%, 98.8%, 99.3%, 99.7%, 99.7 and 100%, respectively.

### 3.3. Molecular Epidemiological Transmission Analysis of Spa t2873 in Farm ‘A’

Farm ‘A’: Dairy herd of approximately 1000 milking cows, Israeli Holstein breed, located in the Negev region in Israel. Cows in Farm A were milked three times a day. Post milking teat disinfection was performed by spraying a commercial approved Iodine. The cows were milked in a rotary parlor in 10 milking groups—in the following order—starting from first lactation, second, third, fourth, and higher lactations. Cows stayed in the same milking group throughout lactation unless they exhibited intramammary infection by a contagious pathogen, e.g., *S. aureus* or *Streptococcus dysgalactiae.* Each milking group consists of 90–120 milking cows. Intramammary-infected (IMI) cows with a contagious pathogen were milked last (named group seven). The additional group included “Dry Cows” (named group 39). Milk sampling was routinely consistent throughout the study and included the following indications: clinically affected quarters, sub-clinical mastitis (above 400,000 somatic cells/mL); 4–7 days post-calving (DIM); before dry-off and post-treatment.

**Figure 1:**
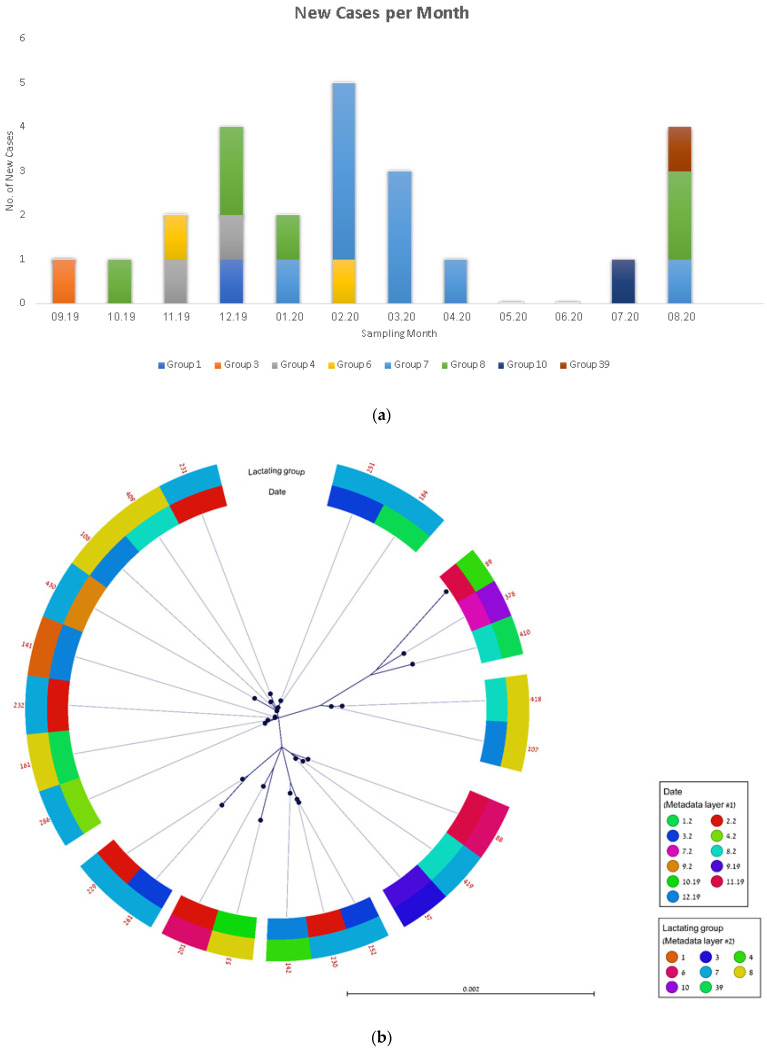
**Transmission analysis of the t2873 strain within farm ‘A’.** (**a**) Epidemic curve of *S. aureus* infection cases, September 2019–August 2020. (**b**) Phylogenetic analysis of 25 *S. aureus* t2873 isolates. Metadata layers include isolation month and milking group.

Twenty-five *spa* t2873 isolates were cultured in Farm A throughout the one-year duration of the study (Figure 1a). The cases appeared for the most part sporadically throughout the year, excluding one cluster of cases in group seven that occurred between January and April 2020. WGS-based analysis was used to analyze the transmission of this strain (see ‘Methods’). The phylogenetic analysis marked according to farm milking group and sampling date is presented in Figure 1b and Figure S1. The pairwise distance between the isolates ranged from 1 to 73 SNP, with a ≤10 SNP distance present in 45 isolate pairs (Figure S1). Within the different milking groups, 17/25 (68%) of cows had direct contact with at least one cow with a closely-related isolate.

The phylogenetic analysis was not able to delineate a clear route of dissemination across time and location within the farm (Figure 1b). However, it was able to identify transmission clusters within and between milking groups (Table 2). For instance, the largest cluster (in purple) that included eight cows was identified in three milking groups one, four, and eight. The IMI group seven served as a destination for the infected cows (e.g., cow 286 from group one; cows 184 and 251 from group two) but also as a focus for further infection and transmission (e.g., cow 141). Through individual tracking, we were able to identify inter-group movements (arrows) that could have resulted in transmission. Such tracking also allowed to explain the dissemination of smaller clusters between milking groups that occurred through an intermediary third milking group: cows 37 and 88 were present together in milking group 39, where the transmission between them might have occurred and passed later to cow 419 that was located in milking group six (with cow 88) before moving to milking group seven.

The order of milking might have played a role in the transmission between consecutive milking groups in two cases: (a) group three was milked after group four and thus might have led to the transmission between cows 142 to 230; (b) group eight was milked after group one and thus might have led to the transmission between cows 141 to 108.

In only one cluster (cluster C, Table 2) no epidemiological connection could be made.

### 3.4. Molecular Features of the Spa t2873 Strain

We analyzed the molecular resistance mechanisms and putative virulence factors of the t2873 strain via WGS. The only resistance gene that was detected was the *tetM* gene, which conferred resistance to tetracycline but not doxycycline (data not shown). The strain also harbored genes for several putative virulence factors, including hemolysins (*hla*, *hlb*, *hld*, *hlgA-B-C*), aurolysin (*aur*), lipase (*lip*), staphopain (*sspB*, *sspC*), intracellular adhesion proteins (*icaA*, *icaB*, *icaC*, *icaD*, *icaR*), collagen adhesion (*cna*), capsule genes (*cap*8A, C-E, G, H-P), Iron-regulated surface determinant (*isd*E-G), *sbi*, Cysteine protease (*ssp*B-C), and Type VII secretion system (*esa*A-C, *ess*A-C, *esx*A-B). 

## 4. Discussion

In continuing with a previous report [3], our survey, on a farm level, showed a substantial prevalence of 27.7% of *S. aureus* (mainly MSSA) on Israeli dairy farms. Other studies from both high- and low-income courtiers [13,14,15] had also reported a substantial prevalence of *S. aureus* intramammary infected cows, but differences in sampling practices and study designs make direct comparisons to our study almost meaningless.

Our study revealed an unexpected phenomenon—we found that a single MSSA strain, t2873, corresponding with ST479 [16], was found in 73.6% of infected farms and was identified in 64.5% of the isolates. This phenomenon is unprecedented and, to the best of our knowledge, was not found in previous national surveys. In a recent survey from Croatia [7], the most common strain (t2678) was identified in only 14% of the isolates, whereas t2873 was found in 5.33%. In a smaller survey from Northern Germany (n = 70) [5], the most common strain was t1403, which was identified in 27.1% of the isolates, whereas t2873 was found in 1.4%. In a survey of *S. aureus* isolates from cattle, pigs, and poultry in Denmark [16], the most common strains were t518 and t524 (22.3%, each). t2873 was found in cattle (4.4%) but not in the other animals. Similarly, in an international study of isolates from Israel, Germany, the USA, and Italy, the ST479 strain (corresponding with t2873) was found in cows but not in goats or sheep [17]. 

What are the possible explanations for the phenomenon that was found in our study? The genomic characterization of the t2873 strain revealed an array of possible virulence factors, most notable intracellular adhesion proteins, hemolysins, and collagen adhesion (*cna*). The role of specific virulence factors in bovine mastitis is not clear. A host-related predilection for specific genes and *S. aureus* clones in bovine vs. goats and sheep has been reported [17]. However, a recent genomic study that looked into the correlation between putative virulence factors to symptomatic bovine mastitis [6] did not find an association between the number or identity of these genes to clinical mastitis. Another genomic study that analyzed the correlation between clinical presentation (clinical vs. subclinical mastitis) with specific genes and clones did not find an independent correlation for genes, but certain clones were associated with clinical infections [18] among them, CC479 (which includes the t2873 strain). Together, these data suggest that the t2873 can be associated with significant bovine disease but not to the extent found in Israeli farms. Furthermore, the genomic data are insufficient to explain the molecular mechanisms that stand behind this phenomenon. A possible epidemiological explanation for this phenomenon is the transmission of this strain between different farms. Such transmission may occur via the purchasing of calves (in general, older cows are not transferred between farms) from infected to non-infected farms. Then, the newly introduced *S. aureus* carrying calf may transmit the bacteria to other animals within the same farm. This possibility was not explored in our study but should be sought as it offers potential prevention opportunities.

The second most common *S. aureus* strain in our study was the t529 type, which was identified in 22 farms and isolates. This strain was reported as a common cause of bovine mastitis [5,7,17]. MRSA was found scarcely (13/440, 3%), most commonly of the t011type (n = 11), a part of the CC398 livestock-associated MRSA clone.

The use of WGS allowed us to analyze the transmission of the t2873 strain within farm ‘A’. Based on the epidemiologic curve (Figure 1a), we concluded that the intramammary infection by this strain was probably longstanding, and thus, it was impossible to trace its origin. However, the use of WGS enabled us to understand the transmission modes—almost all transmission events occurred within the same milking cows group, and inter-group transmission was explained by the transfer of cows amongst the milking groups or due to consecutive milking. Although WGS was used to determine the interspecies transmission of MRSA [19,20], to the best of our knowledge, our study is the first to use this method to analyze the transmission modes within a large dairy cows farm.

In conclusion, our study revealed the dissemination of a single MSSA strain, t2873, to the majority of Israeli dairy cow farms. This strain appears to be highly adapted to this biological niche as it has spread to dairy farms throughout Israel. Hence the possibility of inter-farm transmission via the transfer of calves should be monitored and prevented.

## Figures and Tables

**Table 1:** **Distribution of the *S. aureus spa* types across Israel.**^1^ I-individual sample, T-bulk-tank sample. Other *spa* type that were present in only one sample included 111, 701, 1534, 2346, 2802, 2876, 19691, 19692 and non-typeable.

Spa Type	Farms (n)	Isolates (n)	Sample Type ^1^ (n)
11	3	11	I (10), T (1)
34	6	9	I (7), T (2)
127	2	2	I (1), T (1)
355	2	3	I (3)
529	22	46	I (36), T (10)
543	1	9	I (9)
937	2	2	I (2)
1250	1	13	I (13)
2873	112	284	I (259), T (25)
2970	1	5	I (4), T (1)
9303	12	34	I (32), T (2)
11253	1	7	I (7)
16822	2	2	I (1), T (1)
19690	2	2	I (2)
19693	1	2	I (2)

**Table 2:** **Individual acquisition analysis of the t2873 strain within farm ‘A’.** For each infected cow, the date, location (milking group) and molecular cluster are presented as well as the order of isolation within each cluster. The presumptive source was analyzed based on shared molecular cluster, time and place, except for the first cow within each cluster.

Cow Number	Isolation of *S. aureus*				Presumptive Source	
	Date	Location	Cluster	Order	Location	Cow Number
37	22.09.19	3	A	1	39	-
88	24.11.19	6		2	39	37
419	17.08.20	7		3	7	88
53	23.10.19	8	B	1	6	-
201	04.02.20	6		2	6	53
89	24.11.19	4	C	1	Unknown	
378	13.07.20	10		2	Unknown	
410	04.08.20	39		3	Unknown	
107	10.12.19	8	D	1	8	-
418	17.08.20	8		2	8	107
108	10.12.19	8	E	1	1	141
141	30.12.19	1		2	4	161
161	14.01.20	8		3	4	141
					8	108
231	19.02.20	7		4	7	141/232
232	19.02.20	7		5	7	141/231
286	14.04.20	7		6	1	141
					7	231/232
409	04.08.20	8		7	8	161
430	06.09.20	7		8	7	286
142	30.12.19	4	F	1	4	-
230	19.02.20	7		2	3	142
252	08.03.20	7		3	7	230
184	26.01.20	7	G	1	2/7	-
251	08.03.20	7		2	2/7	184
229	19.02.20	7	H	1	1/7	-
261	12.03.20	7		2	1/7	261

## Data Availability

The data presented in this study are available on request from the corresponding author.

## Supplementary Materials

The following supporting information can be downloaded at: https://www.mdpi.com/article/10.3390/vetsci9070371/s1, Figure S1: SNP distance matrix of t2873 isolates from farm ‘A’.
