# Supplementary material for: The Dissemination of a Single Staphylococcus aureus Strain, Spa-t2873, as the Predominant Cause of Bovine Mastitis in Israeli Dairy Farms
Source: Vet Sci. 2022 Jul 19;9(7):371. doi: 10.3390/vetsci9070371 (PMC9317514; doi:10.3390/vetsci9070371)
Supplement: Supplementary file 1 [file vetsci-09-00371-s001.zip › vetsci-1785851-supplementary.pdf]

Figure S1. SNP distance matrix of t2873 isolates from farm 'A'.

		1	2	3	4	5	6	7	8	9	10	11	12	13	14	15	16	17	18	19	20	21	22	23	24	25
107_S1_L001	1	0	17	16	37	18	18	36	39	39	22	16	20	40	46	19	31	29	20	32	3	31	22	45	32	51
108_S5_L001	2	17	0	3	24	5	1	22	26	26	5	3	3	27	31	6	42	16	3	43	20	18	7	31	19	62
141_S6_L001	3	16	3	0	23	4	4	22	25	25	8	2	6	26	34	5	41	15	6	42	19	17	10	31	18	61
142_S7_L001	4	37	24	23	0	25	25	25	28	8	29	23	27	9	37	26	44	18	27	45	40	20	31	34	21	64
161_S2_L001	5	18	5	4	25	0	6	24	27	27	10	4	8	28	36	5	43	17	8	44	21	19	12	33	20	63
184_S8_L001	6	18	1	4	25	6	0	23	27	27	6	4	2	28	32	7	43	17	4	44	21	19	8	32	20	63
201_S9_L001	7	36	22	22	25	24	23	0	27	27	27	22	25	28	35	25	43	17	25	44	39	19	29	21	20	63
229_S10_L001	8	39	26	25	28	27	27	27	0	30	31	25	29	31	13	28	46	20	29	47	42	22	33	36	23	66
230_S5_L001	9	39	26	25	8	27	27	27	30	0	31	25	29	1	39	28	46	20	29	47	42	22	33	36	23	66
231_S11_L001	10	22	5	8	29	10	6	27	31	31	0	8	8	32	36	11	47	21	8	48	25	23	12	36	24	67
232_S12_L001	11	16	3	2	23	4	4	22	25	25	8	0	6	26	34	5	41	15	6	42	19	17	10	31	18	61
251_S13_L001	12	20	3	6	27	8	2	25	29	29	8	6	0	30	34	9	45	19	6	46	23	21	10	34	22	65
252_S14_L001	13	40	27	26	9	28	28	28	31	1	32	26	30	0	40	29	47	21	30	48	43	23	34	37	24	67
261_S6_L001	14	46	31	34	37	36	32	35	13	39	36	34	34	40	0	37	53	29	34	54	49	31	34	44	32	73
286_S15_L001	15	19	6	5	26	5	7	25	28	28	11	5	9	29	37	0	44	18	9	45	22	20	13	34	21	64
378_S16_L001	16	31	42	41	44	43	43	43	46	46	47	41	45	47	53	44	0	36	45	23	34	38	47	52	39	38
37_S1_L001	17	29	16	15	18	17	17	17	20	20	21	15	19	21	29	18	36	0	19	37	32	6	23	26	7	56
409_S7_L001	18	20	3	6	27	8	4	25	29	29	8	6	6	30	34	9	45	19	0	46	23	21	10	34	22	65
410_S8_L001	19	32	43	42	45	44	44	44	47	47	48	42	46	48	54	45	23	37	46	0	35	39	48	53	40	43
418_S9_L001	20	3	20	19	40	21	21	39	42	42	25	19	23	43	49	22	34	32	23	35	0	34	25	48	35	54
419_S10_L001	21	31	18	17	20	19	19	19	22	22	23	17	21	23	31	20	38	6	21	39	34	0	25	28	9	58
430_S11_L001	22	22	7	10	31	12	8	29	33	33	12	10	10	34	34	13	47	23	10	48	25	25	0	38	26	67
53_S2_L001	23	45	31	31	34	33	32	21	36	36	36	31	34	37	44	34	52	26	34	53	48	28	38	0	29	72
88_S3_L001	24	32	19	18	21	20	20	20	23	23	24	18	22	24	32	21	39	7	22	40	35	9	26	29	0	59
89_S4_L001	25	51	62	61	64	63	63	63	66	66	67	61	65	67	73	64	38	56	65	43	54	58	67	72	59	0
